# History of the Origin and Transactions of the Medicial Societies of Buffalo

**Published:** 1861-11

**Authors:** Thomas F. Rochester


BUFFALO

Medical and Surgical Journal

AND REPORTER.

VOL. I. NOVEMBER, 1861. NO. 4.

### ORIGINAL COMMUNICATIONS.

ART. I.—*History of the Origin and Transactions of the Medical Societies of Buffalo, by* THOMAS F. ROCHESTER, M. D.

(CONCLUDED.)

Dr. Miner reported a severe case of traumatic tetanus, occurring sixteen days after an injury of the hand, resulting in recovery. "Tonics, stimulants and sustaining diet with anodynes, constituted the main reliance." "During the severity of the disease, two grains of acetate of morphia, every four hours in injection, seemed to be attended by the happiest effects." The disease persisted for thirty-three days—chloroform was only administered once.

Dr. White, then gave the history of an instance of idiopatic tetanus, in a child seven years old, also resulting in recovery after a duration of thirty-one days. Quinine, valerinate of zinc, morphine and chloroform, were severally administered, but were finally abandoned, and nutritious diet alone was adhered to. No better illustrations of the good effects of sustaining measures in assisting nature could be cited.

*September 7th*, 1858.—Dr. Rochester reported a case of complete dislocation of the sternal extremity of the clavicle, the patient being present for inspection. The accident was produced by the driver, on a load of wood, being caught between the wood and a transverse beam, under which he attempted to pass. The separated end of the clavicle was thrown upward and forward, and pressed so strongly upon the pomum adami, that breathing and articulation were both difficult. The reduction of the bone was easily effected, and with an apparatus for fractured clavicle, was so far retained in place, that the man recovered good use of the arm, although there remained a decided projection and much mobility at the sterno-clavicular articulation.

Prof. White, reported a case of inversion of the uterus of over fifteen years duration, which he had seen and diagnosed fourteen days after its occurrence. The patient was then seventeen years old, and had completed her second pregnancy. Encouraged by his recent successes in correcting this accident, he had attempted, and with success, the restoration of this long standing case. The patient was placed under the influence of chloroform, and in fifty minutes, the uterus was placed in its normal position. No untoward symptoms succeeded, and at the end of eight days, the patient regarded herself as well. On the ninth day, returning to her room, from which she had walked, she was seized when straining at stool with a violent pain in the epigastrium ; this was speedily followed by the symptoms of general peritonitis, with which affection the patient died on the sixteenth day after the reposition of the uterus. A post-mortem examination, conducted in the presence of several practitioners, most of whom had also been present at the operation, disclosed general peritonitis, most intense, however, in the epigastric region. The uterus, bladder and vagina, were minutely inspected, and no trace of laceration or other injury could be detected, and so complete was the restitution of the former organ, that it could not have been distinguished from the healthy womb of a woman who had borne children. From the autopsy and condition of the patient prior to the ninth day of her convalescence, it is inferred that the supervention of peritonitis was an unfortunate accident, and not a legitimate sequence of the uterine restoration.

*October 5th.*—Dr. Gould made some interesting remarks upon hæmorrhoidal tumors and their treatment, recommending the direct application of nitric acid, undilute, giving an account of three very severe cases successfully treated by this method.

Dr. Butler reported, an instance of rupture of the uterus, eventuating in death. A coroner's examination was held. It appeared that a midwife detected an arm presentation. A physician was sent for, who brought down the feet and delivered. The physicians who conducted the post-mortem examination, and who had also heard the evidence respecting the course and character of the labor, expressed the opinion—"that rupture occurred at the time of turning—**** but no blame could be charged to the medical attendant, as the accident might occur to any person." At the close of the meeting, resolutions were passed respecting the frequency and criminality of "criminal abortions"—also, appointing a committee to confer with the city and county authorities, and to invite the co-operation of the medical societies and associations in this state, in any measure which may be deemed necessary and expedient to lessen these horrible offences against the morality of the community. The Chair appointed Drs. White, Wilcox and Flint Jr., such Committee.

*February* 1*st*, 1859.—Dr. Miner read a report on Tetanus, of much statistical value. He also elaborated the remarks which he had made in a former report of cases of this affection.

Dr. Jansen reported a case of gonorrhea in a male child four years of age, and several members testified to undoubted instances of this disease in children of most tender years, of both sexes. Remarks were also made upon infantile vaginitis and leucorrhea.

*March* 1*st*, 1859.—Dr. Gould gave an account of a post-mortem examination of a man thirty-three years of age, who had typhus fever one year preceeding his death. The brain was softened, the liver weighed twelve pounds and twelve ounces, and the spleen six pounds and twelve ounces. Attention is called to the enormous augmentation of the viscera.

*April* 5*th*, 1859.—Dr. Hawley mentioned that he had succeeded in introducing a catheter into the trachea of a child nine months of age, ill with membraneous croup, through this he injected a strong solution of nitrate of silver. Neither the catheter nor the solution, aggravated the dyspnea, nor did they afford relief, the infant dying about sixty hours after its seizure.

*April* 12*th*, 1859.—Annual Election; President—JAMES M. NEWMAN; Vice President—THOMAS F. ROCHESTER; Secretary—AUSTIN FLINT, Jr.; Treasurer—C. B. HUTCHINS; Librarian—BENJAMIN H. LEMON.

The retiring President, Dr. C. C. Wyckoff, then delivered an address, upon "Chorea Sancti Viti," commending in addition to general hygienic treatment, the employment of arsenic in full doses as an unfailing specific. Appending the history of three cases, under his charge, as corroborating the efficacy of this potent agent.

*April* 19*th*.—The Association extended an invitation to the members of the profession in the city and county, to meet at their rooms, and examine and witness the cardiac phenomena, presented in the person of Mr. E. A. Groux, a congenital fissure of the sternum, affording extraordinary facilities for physical exploration.

Extended remarks on the case by Prof. Austin Flint, are published in the *N. Y. Monthly Review and Buffalo Medical Journal*, Vol. 15, No. 1.

*June 7th*, 1859.—Prof. Rochester read a report on re-vaccination, with a tabular statement of one hundred and thirty-seven cases. The necessity of this procedure was illustrated by the fact, that more than one half were susceptible. Dr. Rochester also urged the employment of recent lymph in place of the generally used crust, insisting and demonstrating, from the table, that the former was more reliable than the latter. A case of traumatic tetanus, successfully treated by free blood-letting, followed by anodynes and stimulants, was reported by Dr. Sylvester Rankin.

*August 2d*, 1859.—Dr. Storck gave a detailed account of the illness of a girl twelve years of age, who to avoid work, swallowed pins to make herself sick. After repeated trials at intervals of several weeks, she succeeded in her novel attempt, sufficiently to cause medical advice to be sought.—After taking morphine, demulcent and farinaceous food and castor oil, she voided by stool *nineteen pins* and *five needles*.

*September 6th*.—Dr. White exhibited a congenital fibro cartilaginous tumor removed by torsion from the pharynx of a child six years of age. Dr. White also reported the removal of the lower segment of the uterus in two instances, for cauliflown excrescence, by the ecraseur. Both operations were successful, "but the disease is unquestionably malignant," and is liable to return.

Dr. Hamilton expressed the opinion, "that a person during sleep cannot be anæsthetised by chloroform." This operation gave rise to some discussion, most of the members coinciding with the view of Prof. Hamilton.—Dr. Wyckoff alone positively dissenting. On this point the writer takes the liberty of stating, that he has recently, with the greatest facility, twice completely anæsthetised a sleeping child, with a much less amount of chloroform than was required by the same patient when awake. The child, a stout boy of four years, had fracture of the forearm.

*October 4th*.—Dr. Rochester reported the successful treatment of several cases of dropsy following scarlet fever, associated with albuminuria, by the internal administration of tannin, also, an instance of severe neonate umbilical hæmorrhage, instantly and effectually controlled by the application o the solution of perchloride of iron.

*Wednesday, November 2d.*—Dr. Newman, the President of the Association, being about to remove from the city, presented his resignation and delivered an earnest and eloquent farewell address.

*December 6th.*—Dr. Hamilton introduced Dr. W. R. Scott, an old and honored practitioner of the city, for some years retired from the profession, yet in his 72d year, possessed of so firm a nerve and an eye so undimmed, that he was daily in the habit of executing the art of caligraphy in a manner both wonderful and pleasing. Dr. Scott stated that he practiced this exercise for the purpose of preserving and improving his vision. He then presented for inspection 1391 words, written upon a circular card 57-100 of an inch in diameter, each letter was beautifully and distinctly formed.—Those curious in such matters will find a special description of the subjects embraced in this minute space, and of the ingenious mode of procedure in the *N. Y. Monthly Review and Buffalo Medical Journal*, vol. 15, pp. 544, 545.

*January 3d,* 1860.—A communication was received from Dr. James M. Newman, the late President of the Society, thanking them for the complimentary resolutions passed at the last meeting, and for the valuable gift of a complete set of obstetrical instruments, the donation of members of the Association.

*March 6th,* 1860.—Prof. White reported a case of gross ignorance and malpractice on the part of a homeopathic quack, enjoying a popular reputation. He had himself, and with the assistance of others, forcibly held back the head of a child in a case of labor, mistaking the caput succedaneum for a nondescript uterine tumor. For more than thirty hours, the poor mother had been thus *compelled* to suffer, and her life additionally doubly imperilled by an enormously distended bladder.

*April 3d,* 1860.—Election—The following officers were chosen :—President—Thomas F. Rochester ; Vice President—C. C. F. Gay ; Secretary—Wm. Treat ; Treasurer—J. F. Miner ; Librarian—Wm. Ring ; Primary Board—F. H. Hamilton, S. B. Eastman, Henry Nichell.

*June 5th,* 1860.—Dr. Miner reported a case of deafness, blindness and ulceration of the cornea, dating three months after parturition, and apparently the effect of lactation, although the patient was young and not unusually feeble.

*September 4th.*—By special appointment Dr. Cronyn opened the discussion on cholera infantum, set down for this meeting. After speaking of the nature and grades of the affection, he stated that where called in season, he had always succeeded in controlling the disease, by the administration of dilute sulphuric acid and laudanum—five drops of the former, with half a drop of the latter, to a child one year of age, repeated at short intervals until vomiting was quieted. Calomel, opium, acetate of lead, tannin and quinine, should subsequently be given, according to the various conditions indicating their applicability—fresh air, warmth, stimulation and diet being never lost sight of. Dissent, as to the good effects of sulphuric acid and laudanum was expressed by Dr. Rochester ; he had made trial of it several years ago by the advice of Dr. Nelson, formerly of this city.—Most of the members present were in the habit of giving calomel and opium, and using external and internal stimulation.

Dr. Strong thought highly of bismuth in large doses. Dr. Treat commended brandy and toast water, with the use of Castillon's powders—(see Testa Præparata, U. S. D.)—boiled in milk. This last was objected to by several, as they usually found it necessary to altogether proscribe milk for a time even in nursing children. For this most natural aliment various substitutes were mentioned, among them raw beef, beef essence and white of egg, mingled with water.

*October 3d*, 1860.—Dr. Treat, on the special subject for the evening, read a most able and interesting paper on diptheria—many of his views were novel and striking—he illustrated very happily the sometimes local and sometimes general effect of the disease, by comparing it to chancre local, and syphilis its frequent constitutional sequence. This paper is placed on file, by order of the Association. It is written illegibly in pencil, it is hoped that both, on account of its intrinsic merit, and as one of many pleasing mementos of its lamented author, that it may soon be revised and given a more enduring form and record.

At this point the writer finds it necessary to conclude the history of the Medical Societies of Buffalo. The abstract of the *proceedings* is carried nearly up to the time, (April, 1861,) assigned to him by his confreres.—He is aware that in the performance of his task, he has for want of time, been compelled to omit much of interest, but he trusts that the selections he has made, have answered the original purpose of the paper, viz : to show by the records of the past, the usefulness and advantages of medical association ; and to urge an earnest and faithful continuance of support to an already honored and respected society—The Buffalo Medical and Surgical Association. One painful duty must not be omitted. The present Association has been singularly blessed in that it has been deprived of so few of its members by death. To others has been deputed the mournful task of commemorating their virtues, but the writer may assist, by mention of their names, to keep green the memory of

MORGAN G. LEWIS,
JAMES M. NEWMAN, and
WILLIAM TREAT.

Over the last of whose grave, the sods of the valley have yet hardly closed, since they opened their bosom to kindly embrace one who in "angel voices" has told us, "the grave is not deep—it is the shining tread of an angel that seeks us."*

*To the above list we must add the name of Bryant Burwell, M. D., who died Sept. 8th, 1861, aetat 65. Dr. Burwell was one of the pioneers in establishing a Medical Society in Buffalo. For many years he had been compelled, by ill-health, to retire from the active duties of his profession. His funeral was largely attended by his medical brethren, and by the citizens generally. Suitable resolutions were passed by the members of the Erie County Medical Society, and a committee was appointed to write a fitting biography—of one who had long held a prominent place in the esteem of all who knew him. T. F. R.

